# Identifying Small Molecules That Promote Quasipalindrome-Associated Template-Switch Mutations in *Escherichia coli*

**DOI:** 10.1534/g3.120.401106

**Published:** 2020-03-27

**Authors:** Julie A. Klaric, Eli L. Perr, Susan T. Lovett

**Affiliations:** Department of Biology and Rosenstiel Basic Medical Sciences Center, Brandeis University, Waltham, MA 02454-9110

**Keywords:** quasipalindrome, replication inhibitor, mutagenesis, screening, small molecules, fluoroquinolones

## Abstract

DNA can assemble into non-B form structures that stall replication and cause genomic instability. One such secondary structure results from an inverted DNA repeat that can assemble into hairpin and cruciform structures during DNA replication. Quasipalindromes (QP), imperfect inverted repeats, are sites of mutational hotspots. Quasipalindrome-associated mutations (QPMs) occur through a template-switch mechanism in which the replicative polymerase stalls at a QP site and uses the nascent strand as a template instead of the correct template strand. This mutational event causes the QP to become a perfect or more perfect inverted repeat. Since it is not fully understood how template-switch events are stimulated or repressed, we designed a high-throughput screen to discover drugs that affect these events. QP reporters were engineered in the *Escherichia coli lacZ* gene to allow us to study template-switch events specifically. We tested 700 compounds from the NIH Clinical Collection through a disk diffusion assay and identified 11 positive hits. One of the hits was azidothymidine (zidovudine, AZT), a thymidine analog and DNA chain terminator. The other ten were found to be fluoroquinolone antibiotics, which induce DNA-protein crosslinks. This work shows that our screen is useful in identifying small molecules that affect quasipalindrome-associated template-switch mutations. We are currently assessing more small molecule libraries and applying this method to study other types of mutations.

Errors in DNA replication and repair can result in genome instability, which can lead to cell death or carcinogenesis. Inverted repeats can form secondary structures that hinder DNA replication. One type of repeated DNA sequence is an imperfect inverted repeat, or quasipalindrome (QP), often found as mutational hotspots (Sherman *et al.* 1974; Ripley 1982; Viswanathan *et al.* 2000; Yoshiyama and Maki 2003).

Mutations in QP regions result from template-switch events during DNA replication. Template switching occurs when DNA polymerase stalls at a QP region and the nascent strand switches to using itself as the template for DNA synthesis. The polymerase and nascent strand switch back to using the correct template strand after incorporating mutations that result in the QP becoming a palindrome with increased complementarity (Ripley 1982). This mechanism of template switching at QP sites was supported through genetic analysis of a natural mutational hotspot in the *thyA* gene in *E. coli* (Dutra and Lovett 2006). The association between QP sites and template-switch mutagenesis was first observed in the yeast *CYC1* gene and bacteriophage T4 *rIIB* gene by Ripley (1982). Since then, template-switch events at QPs have been observed to cause mutagenesis in various contexts, such as in *S. cerevisiae* during double-stand break repair (Hicks *et al.* 2010), *in vitro* when replication is perturbed at common fragile sites (Walsh *et al.* 2013), and in a spectrum of mutations that inactivate the human *TP53* gene (Greenblatt *et al.* 1996).

Considering that template-switch associated mutations are a significant subset of mutational events that have not been thoroughly investigated, in this study, we tested the effect of drugs on their ability to promote template-switch events. Our previous work has shown that azidothymidine (zidovudine, AZT) and other antiviral chain-terminators, as well as drugs known to stall replication through the formation of DNA-protein crosslinks (DPCs), such as 5-azacytidine (5-azaC), stimulate QPM (Seier *et al.* 2012; Laranjo *et al.* 2018). To discover more mutagens for template-switch events, we designed a disk diffusion test for screening small molecule libraries. We utilized the NIH Clinical Collection of small molecules and found 11 positive hits that stimulate template switching. One of the hits was AZT and the other 10 hits were fluoroquinolone antibiotics. As mentioned above, AZT has been shown to promote quasipalindrome mutagenesis (QPM) by stalling the replication fork (Seier *et al.* 2012) through its action as a DNA chain terminator (Olivero 2007; Cooper and Lovett 2011). Fluoroquinolone antibiotics are type II topoisomerase poisons, which target gyrase and topoisomerase IV in bacteria. These antibiotics form DPCs by trapping the cleaved-complex intermediates when the topoisomerases try to relieve DNA supercoils during DNA replication (Drlica and Zhao 1997; Drlica *et al.* 2009).

To validate our screening results, we conducted small-scale disk diffusion assays and fluctuation analysis with fresh stocks of drugs. We tested AZT, 5-azaC, two fluoroquinolone antibiotics, ciprofloxacin (Cipro) and enrofloxacin (Enro), and a type II topoisomerase ATPase inhibitor, novobiocin (Novo). Novo was chosen as a control since it does not result in a DPC with gyrase or topoisomerase IV, in contrast to the fluoroquinolones (Maxwell 1993; Mustaev *et al.* 2014).

Our results validated our findings from the screen and informed us about the amount of drug potency needed for our approach. Overall, our screening approach is effective in identifying potent small molecules that affect QPM. Even though there must be a certain level of drug potency for this method to detect an effect on mutagenesis, this disk diffusion assay can be used to screen additional drug libraries and applied to study a variety of mutations using different reporters for *lacZ* reversion.

## Material and methods

### Bacterial strains, growth conditions, and media

The two mutational reporter strains used are STL20589 (QP5) and STL20590 (QP6), which are derivatives of *Escherichia coli* K-12 MG1655 (Bachmann 1972). The strains were grown at 37° in Luria broth (LB, Lennox formulation) medium, consisting of 1% Bacto-tryptone, 0.5% yeast extract, 0.5% sodium chloride and, for plates, 1.5% Bacto-agar. Tetracycline (15 µg/mL) was used for genetic selection. Lac^+^ reversion mutants were selected on lactose minimal medium containing 56/2 salts (Willetts *et al.* 1969), 0.2% lactose (Sigma Aldrich, St. Louis, MO, USA), 0.001% thiamine (Sigma Aldrich), and 2% agar (ThermoFisher, Sparks, MD, USA). X-gal (40 µg/mL) and IPTG (0.1 mM) (Both from Gold Bio, St. Louis, MO, USA) were included in lactose selection medium as a visual aid for counting colonies.

### Disk diffusion assay to screen small molecule library

The NIH Clinical Collection is a small molecule library containing 700 compounds that have been used in human clinical trials. For more information about the NIH Clinical Collection, contact the National Center for Advancing Translational Sciences (NCATS) Early Translation Branch (ETB@mail.nih.gov). The collection provided to our laboratory was prediluted in sterile water to a concentration of 80 μM. To test a lower concentration of each compound, a small fraction of the entire library was aliquoted and further diluted to 10 μM.

Lactose X-gal papillation plates, as described in Seier *et al.* 2011, but with a lower lactose concentration of 0.5%, were used to detect reversion mutants. Cultures inoculated from single colonies in 1.5 mL LB medium were grown overnight at 37° with aeration. 100 μL of the culture (∼2 × 10^8^ cells) was spread on the papillation plate. 26 sterile 6.35-mm Whatman No. 1 filter disks (Whatman, Maidstone, UK) were added to each plate. 5μL of each drug of interest was added to its corresponding disk and this volume of drug was enough to soak each disk. Sterile water (ddH_2_O) was used as a negative control and 100 μg/mL 5-azaC was used as a positive control. Plates were incubated at 37° for 1-2 days.

### Mutation rate, mutation frequency, and survival determination

**Figure 1:**
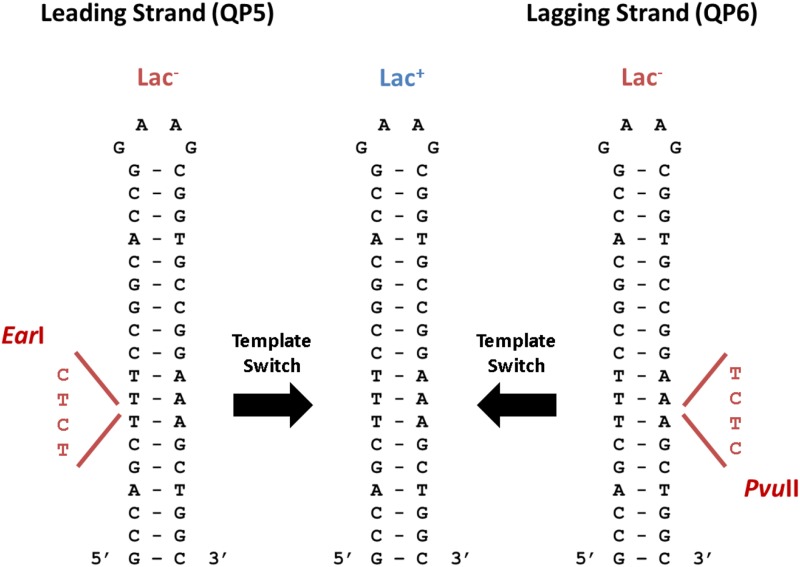
Design of QP mutational reporters in the *E*. *coli lacZ* gene, as described in Seier *et al.* 2011. This set of chromosomal reporters was created by inserting a 4-bp frameshift on both sides of the perfect palindrome, resulting in a Lac^-^ phenotype and creating a leading strand (QP5) and lagging strand (QP6) reporter for QPM. The frameshift causes an *Ear*I restriction site in the QP5 reporter and a *Pvu*II restriction site in the QP6 reporter. An intramolecular template-switch event during leading or lagging strand synthesis results in a Lac^+^ phenotype by removing the 4-bp frameshift, which also causes the loss of the restriction sites.

**Figure 2:**
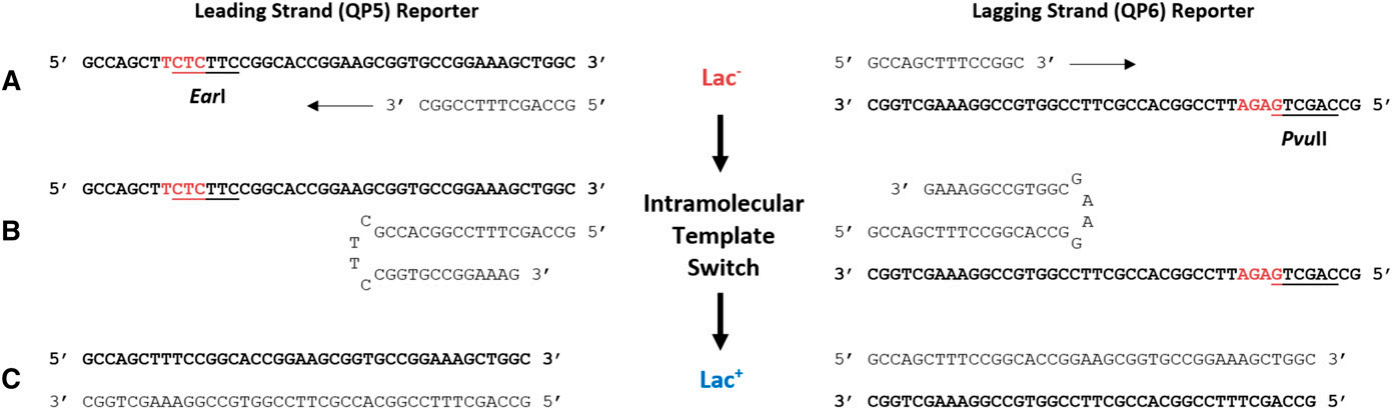
Illustration of reversion of our *lacZ* reporters (QP5 and QP6) through intramolecular template switching. A) The Lac^-^ sequence of the palindrome within *lacZ* is shown with the template strand bolded. The 4-bp frameshift that causes the palindrome to be a quasipalindrome is represented in red. The frameshift results in the creation of a restriction site, which is underlined. B) During DNA replication, the quasipalindrome can form a hairpin structure leading to intramolecular template switching. This results in Lac^+^ reversion and removal of the 4-bp frameshift and restriction site. C) Shown is the Lac^+^ sequence of the palindrome within *lacZ* after the template-switch mutation has been incorporated.

Cultures inoculated from single colonies in 1.5 mL LB medium were grown overnight at 37° with aeration. The culture was diluted 1:100 in fresh 1.5 mL LB medium and grown for 2 hr at 37° with aeration. After this incubation, the entire culture was spun down, and the cell pellet was resuspended in 150 μL of fresh LB medium. The sample was split evenly among tubes with 1.5 mL of LB medium +/− 0.1 μM of the drug indicated in each experiment. The tubes were grown for another 2 hr at 37° with aeration. The entire culture was recovered by microcentrifugation, washed twice with 1 × 56/2 buffer, and resuspended in 200 μL of 1 × 56/2 buffer. 20 μl of the cells was subjected to serial dilution and plated on LB plates to determine the colony forming units (cfu). The remaining cells were plated on minimal lactose medium containing X-gal and IPTG (LacMinXI) and incubated for 2 days at 37°. For strains with high mutation rates and frequencies, serial dilutions of cultures in 1 × 56/2 were plated on LacMinXI. Mutation rates were determined using the Ma-Sandri-Sarkar maximum likelihood estimator through the FluCalc webpage (Radchenko *et al.* 2018). Mutant frequencies were also calculated and statistical significance (*P* < 0.05) was determined by paired sample *t*-test between untreated and treated samples. Specificity of the QP5 and QP6 reporters for only intramolecular template-switch events was confirmed as described in Seier *et al.* 2011. Colony PCR and restriction digests were conducted on 4 revertants of each strain exposed to no treatment or the various compound treatments (0.1 μM 5azaC (Sigma Aldrich), Novo (Sigma Aldrich), AZT (Sigma Aldrich), Enro (Sigma Aldrich), and Cipro (Sigma Aldrich)) from the mutation assay. For these reporters, quasipalindrome-associated template-switch mutations were characterized by the loss of *Ear*I (QP5) or *Pvu*II (QP6) restriction sites (Figure 1 and Figure 2). In addition, 2-4 revertants from each strain and treatment were sequence-verified (GENEWIZ, Inc.). The Lac^+^ phenotype, with and without drug exposure, was determined to be the result of an intramolecular template-switch event and not due to a different mutational event that restored LacZ function.

### Data availability

Supplementary Table 1 lists the small molecules included in the NIH Clinical Collection used in this study. Supplementary Figure 1 shows the small-scale disk diffusion assay results for two false negatives: nalidixic acid (Sigma) and didanosine (Alfa Aesar). Primer sequences used in this study are found in Seier *et al.* 2011. Strains are available upon request. Supplemental material available at figshare: https://doi.org/10.25387/g3.11771001.

## Results

Quasipalindrome-associated template-switch mutations were detected using reporters in the *E*. *coli lacZ* gene (Figure 1). This set of chromosomal reporters has a 4-bp frameshift mutation on either side of an 18-bp QP. This frameshift mutation results in the Lac^-^ phenotype in strains containing these reporters. Each strain reverts from Lac^-^ to Lac^+^ only after an intramolecular template-switch event occurs, which removes the 4-bp frameshift (Figure 2). Due to their design, these strains report template switching on either the leading (QP5) or lagging (QP6) strand of replication (Seier *et al.* 2011).

**Figure 3:**
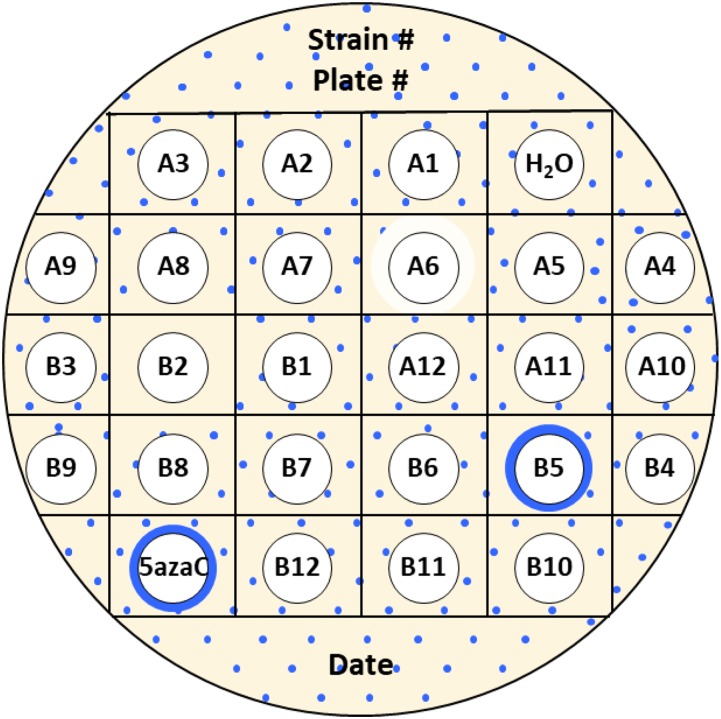
Layout of a papillation plate for screening small molecule libraries using disk diffusion assay. 24 small molecules can be screened on one plate. Distilled water is used as a negative control and 100 μg/mL 5-azaC is used as a positive control. In this example, B5 is a positive hit for inducing mutagenesis since it produces a blue halo around the filter disk. B2 represents a small molecule that decreases mutagenesis since it prevents the formation of blue colonies compared to the negative control. Compound A6 is an illustration of drug that is bactericidal because it produces a zone of inhibition but does not affect mutagenesis.

To identify novel drugs that promote QPM, a disk diffusion assay was developed to screen small molecule libraries using our reporter strains (Figure 3). The medium used in the screen allows Lac^+^ revertants to be visualized as blue, out-growing papillae on a faint white lawn. On one lactose X-gal papillation plate, 24 small molecules can be tested by adding each molecule to its specified filter disk. Only 5 μL of each drug was added to each filter disk to prevent crosstalk between drugs as much as possible, while still allowing proper diffusion. We use distilled water as a negative control and 100 μg/mL (409.5 μM) 5-azaC as a positive control. This concentration of 5-azaC was chosen since it has been shown to induce QPM with the disk diffusion assay (Laranjo *et al.* 2018). If a compound stimulates template switching, after one or two days, a blue halo around the filter disk containing the compound will appear. The presence of blue colonies is the result of the *lacZ* gene becoming functional after a template-switch event. If a compound does not affect template switching, the filter disk will appear the same as the distilled water negative control. If a compound decreases the rate of template switching, there will be a decrease in blue colonies around the filter disk compared to the negative control. Killing by the compound produces a zone of inhibition, a clear region surrounding the disk.

**Figure 4:**
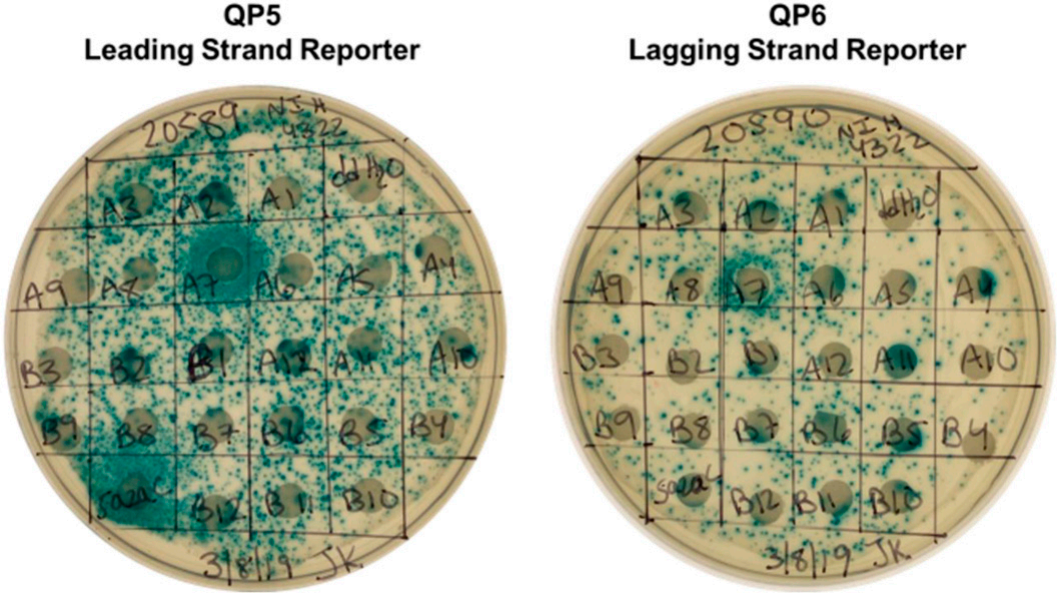
Examples of disk diffusion assays with leading and lagging strand reporters. Blue halos represent increased template switching. In these examples, A7, which is enrofloxacin, was found to stimulate template switching on the leading and lagging strand.

The NIH Clinical Collection, a library containing 700 small molecules (Supplementary Table 1), was utilized in the screen. This library has been used previously to discover anti-coronavirus drugs (Cao *et al.* 2015) and inhibitors of HIV-1 integrase activity (Abrahams *et al.* 2018). The screen was conducted using two different concentrations: 10 μM and 80 μM. Example images of lactose X-gal papillation plates containing the QP5 and QP6 reporter stains and one plate from the NIH Clinical Collection are shown in Figure 4.

Eleven small molecules were found to promote template-switch mutagenesis, as seen in Table 1. One of the hits was AZT, which is a known mutagen of QPM and, therefore, supported the effectiveness of our screen. The 10 other small molecules were all the fluoroquinolones provided in the NIH Clinical Collection. All the hits increased QPM with the leading strand reporter, but only enrofloxacin (Enro) was found to increase template switching on the lagging strand. Various studies have shown that there is a strand bias with QPM (Rosche *et al.* 1997; Yoshiyama *et al.* 2001; Yoshiyama and Maki 2003; Seier *et al.* 2011). This could contribute to the lack of effect of 10 of the hits on the lagging strand reporter. Another possible explanation is that a higher concentration of each drug is needed to see an effect on the lagging strand. Unfortunately, the starting concentration of our library was 80 μM and, thus, it was not possible to repeat the screen at an increased concentration. Also, we did not find any small molecules that prevent QPM in this screen since we did not detect any compounds that decreased the background level of *lacZ* reversion.

**Table 1:** List of small molecules from the NIH Clinical Collection that stimulate QPM and their major mechanism of action. Ten out of eleven hits are fluoroquinolone antibiotics.

	Compound Name	Mechanism of Action
1	Azidothymidine (Zidovudine, AZT)	Thymidine analog; chain terminator
2	Enrofloxacin	Fluoroquinolone antibiotic; type II topoisomerase poison
3	Gatifloxacin	
4	Levofloxacin	
5	Moxifloxacin hydrochloride	
6	Norfloxacin	
7	Ofloxacin	
8	Pazufloxacin	
9	Pefloxacin mesylate	
10	Rufloxacin hydrochloride	
11	Tosufloxacin tosilate	

**Figure 5:**
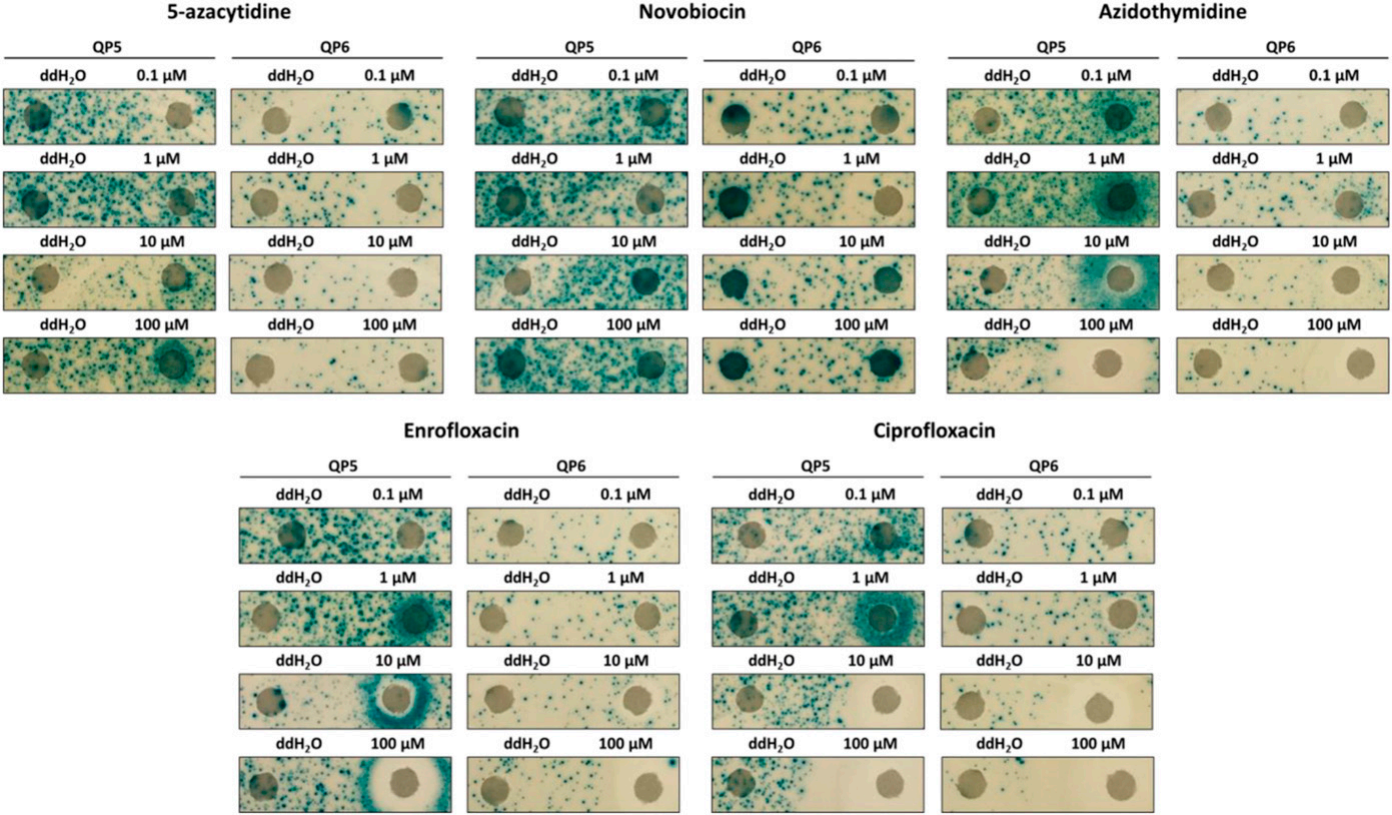
Mutator effect of fresh stocks of 5-azaC, Novo, AZT, Enro, and Cipro. Represented is a disk diffusion assay for Lac reversion using QP5 and QP6 reporters on lactose X-gal papillation medium. Each disk on the right was saturated with 5 μL of specified drug at the indicated concentrations. Each control disk on the left was spotted with 5 μL of distilled water.

Next, the screen’s findings were validated by conducting a disk diffusion assay with fresh stocks of small molecules (Figure 5). AZT, 5-azaC, two fluoroquinolone antibiotics, Cipro and Enro, and a competitive inhibitor of gyrase and topoisomerase IV ATPase activity, novobiocin (Novo), were tested (Sugino *et al.* 1978; Peng and Marians 1993; Lewis *et al.* 1996). Novo is a type II topoisomerase inhibitor that does not result in DPCs in contrast to the fluoroquinolones. Therefore, Novo was chosen to see if the effect of the fluoroquinolones on template switching was due to the formation of DPCs or general inhibition of the type II topoisomerases. Novo did not cause an effect on QPM in this assay, suggesting the effect of the fluoroquinolones on template switching was due to the formation of DPCs. 5-azaC only showed an effect on the leading strand reporter at the highest dosage (100 μM). AZT, Enro, and Cipro caused a large increase in QPM on the leading strand and a mild increase on the lagging strand. The larger zones of inhibition caused by Cipro at 10 μM and 100 μM concentrations compared to those caused by AZT and Enro at those concentrations suggest that Cipro is much more bactericidal.

**Figure 6:**
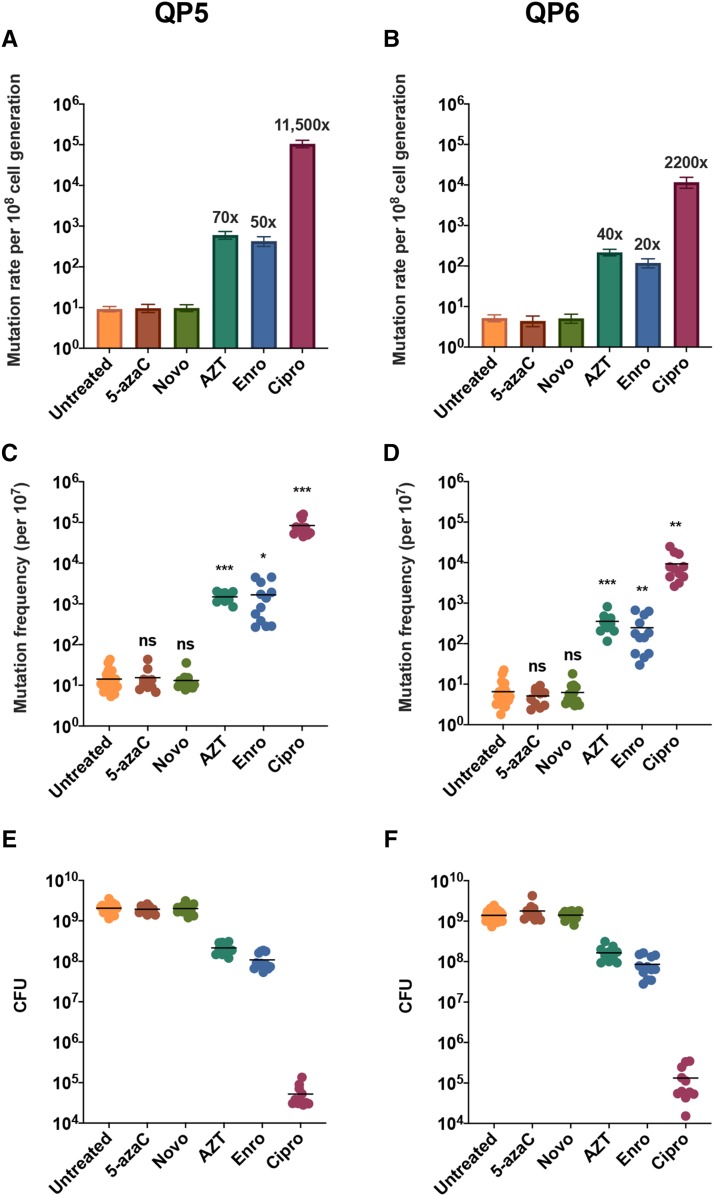
Mutation rates, mutation frequencies, and survival after QP5 (a, c, e) and QP6 (b, d, f) reporter strains were exposed to 0.1 μM of 5-azaC, Novo, AZT, Enro, and Cipro. Paired *t*-test was used to calculate significance between untreated and corresponding treated mutation frequencies. * indicates *P* < 0.05 ** indicates *P* < 0.01 *** indicates *P* < 0.001.

Since disk diffusion assays only provide qualitative data, reversion rates and mutation frequencies with and without acute drug treatment were calculated through fluctuation analysis (Figure 6). Survival is shown to compare the potency of the different drugs used. The assays were conducted with 0.1 μM of each drug because this concentration provided sublethal conditions for Cipro, the drug with the highest potency compared to the others tested. At this concentration, 5-azaC and Novo did not influence QPM. AZT, Enro, and Cipro showed a strong stimulation of template switching on both reporters.

After examining the entire list of small molecules in the collection (Supplementary Table 1), the following four false negatives, which should have stimulated QPM, were identified: 5-azacytidine, nalidixic acid (Nal), stavudine (d4T), and didanosine (ddI). 5-azaC requires at least a concentration of 100 μM to affect template switching (Figure 5). Nal is a quinolone antibiotic and type II topoisomerase poison (Lesher *et al.* 1962; Peng and Marians 1993). d4T and ddI are antiviral DNA chain terminators like AZT and were previously found to stimulate QPM (Mamber *et al.* 1990; Seier *et al.* 2012). Only Nal and ddI were retested using the small-scale disk diffusion test and neither of the compounds increased QPM at the concentrations tested (Supplementary Figure 1). These data suggest that the false negatives found are not as potent for QPM, compared to Enro, Cipro, and AZT.

## Discussion

To understand the mechanism behind widely understudied quasipalindrome-associated template-switch mutations, we designed a disk diffusion test that can be used to screen small molecule libraries to discover compounds that affect these mutations. Using the NIH Clinical Collection, we were able to detect 11 mutagens that stimulate template switching at a QP site. We identified the entire class of type II topoisomerase poisons, the fluoroquinolones, and AZT as potent mutagens for template-switching. Due to the lack of a mutagenic effect by Novo, the effect of the fluoroquinolones is likely to be the result of DNA-topoisomerase II and IV trapped covalent complexes. These results were validated with a small-scale disk diffusion test and fluctuation analysis. Small molecules that prevent QPM were not detected, but we aspire to discover these types of compounds as more chemical libraries are screened.

We identified four false negatives in the library that should have stimulated QPM: 5-azaC, Nal, d4T, and ddI. 5-azaC requires a higher dosage to induce template switching than the 80 μM limit of our collection. Fluoroquinolones are derived from Nal, the first antibacterial quinolone utilized clinically (Lesher *et al.* 1962; Hooper and Jacoby 2016). Fluoroquinolones differ from quinolones by the addition of fluorine at position 6, which significantly increases the antimicrobial potency of the compound (Ito *et al.* 1980; Koga *et al.* 1980; Eliopoulos *et al.* 1984). Nal was not detected because of its low potency as a type II topoisomerase poison.

Like AZT, d4T and ddI have been used as antiretroviral therapies and act as DNA chain terminators. Seier *et al.* (2012) demonstrated that d4T and ddI stimulate QPM, but they required a significantly higher concentration to see an effect compared to AZT. Two other anti-HIV dideoxynucleosides, zalcitabine (ddC) and lamivudine (3TC), are found in the NIH collection and did not enhance template-switch mutagenesis. Previous work in *E. coli* has shown that AZT is significantly more potent at inducing DNA damage than the dideoxynucleosides (Mamber *et al.* 1990). Since AZT and the dideoxynucleosides must be phosphorylated to be active, it has been postulated that AZT might be a better substrate for phosphorylation than the other chain terminators in *E. coli*, which could explain the differences in potency (Mamber *et al.* 1990; Lavie *et al.* 1998).

The limitations of this screening method are that it only produces qualitative data and must be followed up with fluctuation analysis to obtain quantitative data. Also, the detection of a hit relies on the level of potency of the small molecule. The starting concentration of the screening library needs to be high enough to prevent false negatives. Nevertheless, this method is inexpensive and effective at discovering potent mutagens for quasipalindrome-associated template-switch mutations. Future directions involve repeating our screen with other publicly available chemical libraries. Our screening approach will also be applied to find mutagens that stimulate base substitution and frameshift mutations, using reporters already created for *lacZ* reversion assays (Cupples and Miller 1989; Cupples *et al.* 1990; Seier *et al.* 2011).
